# Delayed right ventricular lead perforation complicated by tamponade in biventricular hypertrophic cardiomyopathy

**DOI:** 10.1002/ccr3.1224

**Published:** 2017-10-16

**Authors:** Najla Kourireche, Amal Boutakhrit, Fatima Chikhi, Ibtissam Fellat, Mohammed Cherti

**Affiliations:** ^1^ Cardiology B Department Ibn Sina Hospital Med V University Rabat Morocco

**Keywords:** Hypertrophic cardiomyopathy, lead perforation, pericardial effusion, permanent pacemaker, ventricular perforation

## Abstract

This report highlights the importance of a more cautious approach in a patient with a history of implanted cardiac leads presenting with chest pain or dyspnea, to prevent overlooking cardiac lead perforations especially in hypertrophic cardiomyopathy which seems to be not absolutely protective.

## Introduction

Pacemaker lead perforation is a rare but serious and potentially life‐threatening complication of pacemaker implantation, ranging from 0.1% to 0.8% 1. Lead perforations are recognized mostly during or shortly after implantation, subacute and late perforations are less frequent, and atrial lead is more concerned. We report here a case of delayed right ventricular perforation complicated by a tamponade in a patient with hypertrophic cardiomyopathy that we successfully managed by closed intervention without exposing the patient to surgery.

## Case Report

A 47‐year‐old woman, with a history of familial biventricular hypertrophic cardiomyopathy, underwent dual chamber permanent pacemaker (PPM) for complete atrioventricular block. The right atrial (RA) and right ventricular (RV) leads were implanted, respectively, in the anterolateral wall of the right atrium and the RV apex.

She presented, 45 days later, with acute chest pain that was exacerbated by deep breathing. Chest X‐ray report stated that the intracardiac leads were in place. The PPM interrogation found a normal function of the RV and RA leads. Echocardiography showed minimal pericardial effusion (10 mm) which was related to a postoperative inflammatory reaction. Treatment with ibuprofen was started. The evolution was favorable and symptoms disappeared.

**Figure 1:**
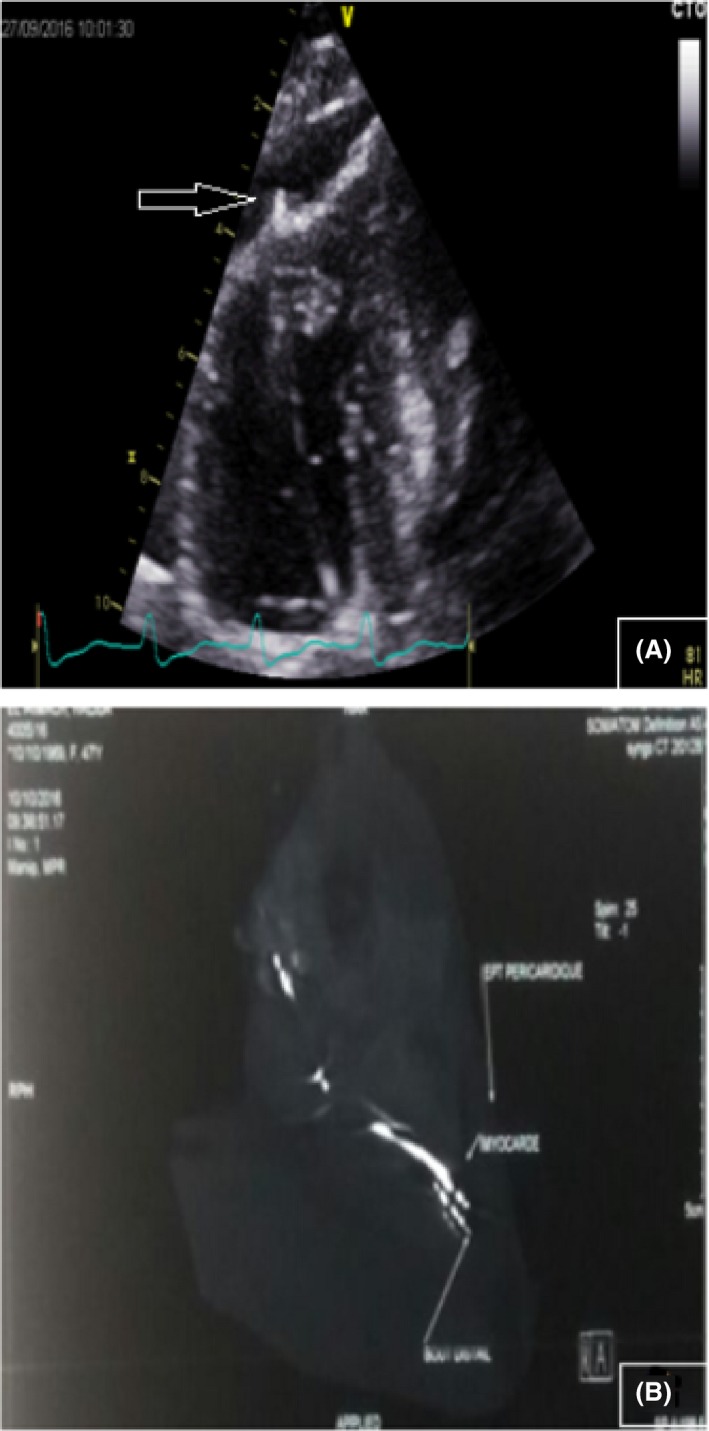
(A) Zoom of right ventricle in the apical four‐chamber view: lead tip visualized in the pericardial space, confirming perforation (arrow). (B) Chest computed tomography showing a myocardial perforation by the pacing lead (arrow). Note the associated pericardial effusion (arrow).

The 4th month echocardiographic follow‐up showed aggravation of the pericardial effusion (20 mm), with no hemodynamic significance. A displacement of the RV lead was suspected (Fig. 1A). Device interrogation found that R wave amplitude and pacing impedance were normal while the pacing threshold passed from 0.7 V to 1.25 V. Chest computed tomography (CT) was performed and confirmed perforation of the right ventricular apex by the RV lead (Fig. 1B).

Three days later, she presented with sudden onset of shortness of breath. Echocardiography showed massive pericardial effusion, with impending subsequent tamponade. An emergent bedside ultrasound‐guided pericardiocentesis was performed successfully using a subxiphoid approach. We decided to manage the patient conservatively. The ventricular lead was extracted and repositioned in the RV septum. Postprocedure echocardiogram confirmed no increase of the thin layer of pericardial effusion. No symptoms of tamponade were observed during or after the procedure in 6 months of follow‐up.

## Discussion

Patients with late lead perforation might have a variety of presenting symptoms 2, 3. In our case, the initial presentation with chest pain and minimal pericardial effusion were disconcerting. Postimplantation pericardial effusion can be caused by a traumatic inflammation of the myocardium or irritation of the visceral pericardium via immune‐mediated mechanisms, but it can result from a lead perforation especially in the presence of risk factors such as age, female sex, and concomitant therapies such as anticoagulant or antiplatelet medication 4. Patients developing postoperative pericarditis should be followed closely due to the risk of cardiac tamponade 5.

Thin heart muscle itself may favor perforation 6. Although a dilated, thin myocardium could be seen as a vulnerable substrate, normal myocardial thickness, or hypertrophy, is not absolutely protective 7. According to published data in the literature, this is the first case of RV perforation in young patient with biventricular hypertrophic cardiomyopathy. Indeed, it is safer to screw the electrode into the ventricular septum than into the apex in patients with hypertrophic cardiomyopathy.

The management question of late lead perforation is whether to extract the lead or not, and if so, whether to extract percutaneously or surgically. Indeed, there is no consensus on the management of displaced leads and the perforations they cause, particularly when they are asymptomatic 8. Surgical intervention seems to be the treatment of choice in the case of hemodynamic instability, rapid progression of pericardial effusion, or if closed pericardiocentesis fails 9. However, as for our case, successful management with closed pericardiocentesis and a pericardial drain in place can be performed, and a lead extraction can be carried out in the electrophysiology laboratory or operating room system with availability of emergency back‐up thoracotomy 10.

## Conclusion

Late RV perforation is a rare but serious complication of PPM implantation. It may be completely asymptomatic or life‐threatening. Hypertrophic myocardium seems to be not absolutely protective. Postimplantation pericardial effusion should be followed closely due to the risk of cardiac tamponade. According to our case, this fatal complication can be successfully managed by closed pericardiocentesis and the RV lead repositioning, but careful hemodynamic and echocardiographic monitoring is necessary because of the risk of delayed retamponade as the site of perforation may not be fully closed.

## Authorship

NK: involved in drafting the manuscript. AB: involved in drafting the manuscript. FCH: involved in critical revision of the manuscript. IB: involved in general supervision and critical revision of the manuscript. MCH: involved in general supervision and critical revision of the manuscript.

## Conflict of Interest

The authors declare that they have no conflict of interests concerning this article.
